# Healing of excisional wound in alloxan induced diabetic sheep: A planimetric and histopathologic study

**Published:** 2013

**Authors:** Siamak Kazemi-Darabadi, Farshid Sarrafzadeh-Rezaei, Amir-Abbas Farshid, Reza Baradar-Jalili

**Affiliations:** 1 *Department of Clinical Sciences, Faculty of Veterinary Medicine, Urmia University, Urmia, Iran;*; 2 *Department of Pathobiology, Faculty of Veterinary Medicine, Urmia University, Urmia, Iran; *; 3 *Department of Surgery, Faculty of Medicine, University of British Columbia, Vancouver, British Columbia, Canada.*

**Keywords:** Alloxan, Diabetic wound, Healing, Sheep, Skin

## Abstract

Healing of skin wound is a multi-factorial and complex process. Proper treatment of diabetic wounds is still a major clinical challenge. Although diabetes mellitus can occur in ruminants, healing of wounds in diabetic ruminants has not yet been investigated. The aim of this study was to evaluate healing of ovine excisional diabetic wound model. Eight 4-month-old Iranian Makoui wethers were equally divided to diabetic and nondiabetic groups. Alloxan monohydrate (60 mg kg^-1^, IV) was used for diabetes induction. In each wether, an excisional wound was created on the dorsum of the animal. Photographs were taken in distinct times for planimetric evaluation. Wound samples were taken on day 21 post-wounding for histopathologic evaluations of epidermal thickness, number of fibroblasts and number of new blood vessels. The planimetric study showed slightly delay in wound closure of diabetic animals, however, it was not significantly different from nondiabetic wounds (*p *≥ 0.05). Furthermore, epidermal thickness, number of fibroblasts and number of blood vessels were significantly lower in diabetic group (*p *< 0.05). We concluded that healing of excisional diabetic wounds in sheep may be compromised, as seen in other species. However, contraction rate of these wounds may not be delayed due to metabolic features of ruminants and these animals might go under surgeries without any serious concern. However, healing quality of these wounds may be lower than normal wounds.

## Introduction

Healing of skin wound is a multi-factorial and complex process.^1^ This process is mainly divided into inflammatory, proliferative, and remodeling phases.^2^ In the proliferative phase, re-epithelialization, angiogenesis, and granulation occur, and endothelial cells, fibroblasts, and keratinocytes are mainly involved.^3^ Re-epithelialization is one of the earliest and most important stages of wound healing mediated by keratinocytes.^4^ Angiogenesis, or the process of forming new blood vessels, is critical to wound healing because it allows the delivery of vital cells and nutrients to the wound,^5^ and is induced by many angiogenic factors releasing from fibroblasts and macrophages.^6^ Fibroblasts not only are responsible for collagen synthesis, but also play an important role in wound contraction by differentiation to myofibroblasts. They also excrete various growth factors and cytokines involved in the wound healing such as keratinocyte growth factor (KGF), vascular endothelial growth factor (VEGF) and transforming growth factor (TGF).^7^

Treatment of chronic wounds, especially in diabetic patients, is a major clinical challenge.^8^ Diabetes mellitus is a disorder of carbohydrate, lipid and protein metabolism that can affect many organs, including skin.^9^ The full mechanisms of impaired wound healing in diabetes mellitus have not yet been demonstrated.^10^ Nonetheless, increased inflammation, disordered extracellular matrix synthesis and remodeling, and re-epithelialization problems are evident in these wounds.^8^ On the other hand, the effect of diabetes on the wound healing is the impairment of cellular proliferation for most cell types.^11^ Furthermore, micro- and macro-angiopathy, and retardation of granulation tissue formation may also be present.^12^ It is suggested that cellular and molecular signals that normally encourage wound healing do not exist in diabetic wounds, and this is the major factor of delayed healing.^13^

Several researchers investigate healing problems of diabetic wounds in various species such as human, rat, mouse, rabbit, and pig.^13^^-^^18^ Although diabetes develops in sheep,^19^ differences between diabetic and nondiabetic wound healing of this species have not yet been evaluated. The aim of this study was to evaluate healing characteristics of ovine excisional diabetic wound model.

## Materials and Methods

**Animals. **All experimental procedures were approved by the Advisory Committee of Urmia University Research Council. Eight 4-month-old Iranian Makoui wethers (average body weight 25 ± 1.5 kg at arrival) were used in this study. The overall health of the wethers was monitored before and throughout the study. The animals were kept in barn and received antibiotic and anti-parasitic medications prior to study and were acclimatized to the experimental conditions for 14 days. Body weight of the animals was recorded at arrival and at end of the study. The animals had free access to hay and tap water throughout the study. Wool of the animals was clipped a week before surgery. Ear tags were used after local infiltrative anesthesia by 1 mL of lidocaine HCl 1% solution (Shahid Ghazi Pharmaceutical Co., Tabriz, Iran). The animals were randomly divided into two experimental groups (n = 4). One group was considered nondiabetic (ND) and did not receive any diabetogenic material and in diabetic (D) group, diabetes was induced by alloxan monohydrate (Sigma Aldrich Co., Dorset, UK).

**Blood glucose and serum insulin assays. **To ensure diabetes induction and maintenance, venous blood samples were taken in the fasted state from external jugular vein three weeks before alloxan injection and throughout the study weekly. Blood glucose levels were determined using glucometer device (On-Call Plus; ACON Biotech Co. Ltd., Hangzho, Zhejiang, China). Serum insulin values were measured using ovine insulin ELISA kit (ALPCO Diagnostics, Salem, NH, USA).

**Diabetic conscious sheep model. **In the diabetic group, 10% solution of alloxan monohydrate was infused at a dose of 45 mg kg^-1^ into external jugular vein, after 24 hr of fasting, similar to procedure performed in dogs.^20^ Alloxan was injected immediately after dissolving in normal saline solution because of its very short half-life in saline and blood. None of the wethers developed diabetes on following two weeks, and another infusion of alloxan at a dose of 60 mg kg^-1^ was performed. Diabetes induction was confirmed a week later. 

**Surgical wound model. **Two weeks after diabetes induction, dorsum of the sheep were shaved and under local anesthesia and aseptic preparation, a square measuring of 2×2 cm was outlined in each animal using a marker. Then, the demarcated areas of skin were removed by scalpel. The wounds were left undressed after hemostasis. Systemic and topical antibiotics were used to prevent infection for 5 days. 

**Fig. 1:**
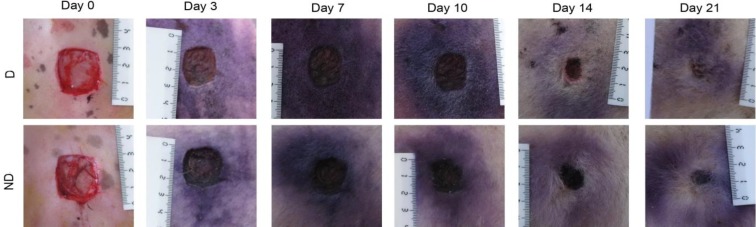
Serial photographs of wounds in diabetic (D) and nondiabetic (ND) sheep in different days.

**Planimetry. **Photographs were taken immediately after wounding and on days 3, 7, 10, 14, and 21 post-operation by a digital camera while a ruler was placed near the wounds (Fig. 1). The wound areas were analyzed by Measuring Tool of Adobe Acrobat 9 Pro Extended software (Adobe Systems Inc, San Jose, CA, USA) and wound contraction percentage was calculated using the following formula:

*Percentage of wound contraction = (A*_0_ *– A*_t_ *) / A*_0_ *× 100*

where A_0_ is the original wound area and A_t_ is the wound area at the time of imaging.^21^

**Histopathologic analysis. **Tissue biopsies were taken under local anesthesia for histopathologic evaluations on day 21 post-operation. The specimens were fixed in 10% buffered formalin solution, dehydrated in alcohol, cleared in xylene and embedded in paraffin. Five μm thick sections were cut and stained with hematoxylin and eosin (H&E). The slides were assessed under light microscope for epidermal thickness, number of fibroblasts, and number of new blood vessels.

The thicknesses of the epidermal areas formed were measured in ten different places along the wound surface by using a graticule eyepiece lens and the average of these areas were taken as the thickness of the epidermis. The number of newly formed blood vessels and fibroblasts in H&E stained sections were counted by special morphometric lens in 0.25 mm^2^ microscopic field. Ten different areas in the sections were counted and mean values were taken into account.

**Statistical analysis. **All experimental results were presented as mean ± standard deviation (SD). Differences were considered significant at *p *< 0.05. Planimetric data were analyzed with repeated measures analysis of variance (ANOVA) using SAS Proc Mixed (Version 9.2, SAS Institute Inc., Cary, NC, USA). The models included the fixed effects of group, treatment, day and their interactions and also a random effect associated with the sheep nested within group. The models were compared using likelihood ratio test statistic. The correlation structure between the repeated measures over time were examined by including SP (POW), SP (GAU), and SP (SPH) to the model and the appropriate covariant structure was selected based on the lowest value of Akaike information criterion (AIC) statistic. After fitting the model, assumptions of normal distribution, equal variance and unusual observations were checked using the residuals. If a significant fixed effect was detected, differences between least squares means were compared using the Bonferroni test. Insulin and glucose concentrations were analyzed by one-way ANOVA and Tukey’s test to assess statistical differences between experimental groups, and histopathologic parameters were analyzed by Student’s *t*-test.

## Results

Table 1 shows blood glucose and serum insulin concentrations in the wethers before and after induction of diabetes. The results showed significant increase in glucose and decrease in insulin concentrations in diabetic group after administration of alloxan monohydrate and confirmed induction of diabetes (*p *< 0.05). The effect of diabetes on body weight of the animals is shown in Table 2.

**Fig. 2:**
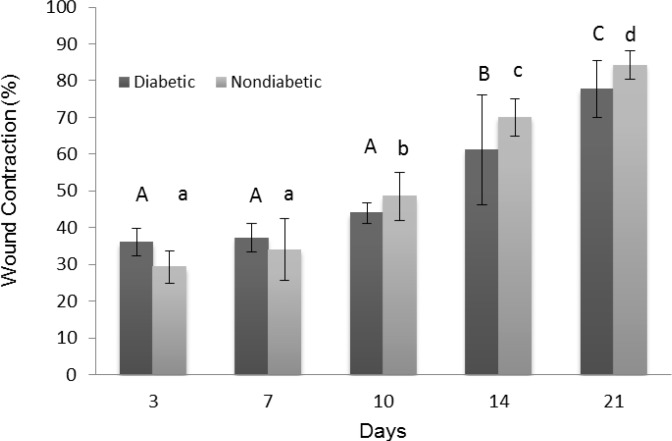
Wound contraction percentage (Mean ± SD) in different time points compared to first day of wounding.

^ABC^ different capital letters indicate significant differences (*p *< 0.05) in wounds of diabetic sheep in different time points.

^abcd^ Different minuscule letters indicate significant differences (*p* < 0.05) in wounds of nondiabetic sheep in different time points.

Wound contraction percentage in different groups over time series is shown in figure 2. Although healing rate of diabetic wounds were slightly lower than nondiabetic wounds after day 7, there was not any significant difference between diabetic and nondiabetic wound contraction percentages (*p *≥ 0.05). However, the figure shows that time has significant effect on wound contraction of all wounds (*p *< 0.05).

**Table 1:** Concentration (Mean ± SD) of serum insulin and blood glucose before and after injection of alloxan monohydrate.

	**Nondiabetic**	**Diabetic ** **(pre-diabetic)**	**Diabetic** **(post-diabetic)**
**Insulin (µIU mL** ^-1^ **)**	13.19 ± 1.13^a^	12.34 ± 1.14^a^	2.08 ± 1.10^b^
**Glucose (mg dL** ^-1^ **)**	61.83 ± 7.03^a^	64.41 ± 9.85^a^	199.41 ± 56.97^b^

ab Means in the each row followed by the same letter are not significantly different (*p *≥ 0.05).

**Table 2:** Body weight of animals (Mean ± SD) recorded at arrival and end of the study.

	**Arrival (kg)**	**End of the Study (kg)**
**Nondiabetic**	24.50 ± 2.08	27.00 ± 2.58
**Diabetic**	25.25 ± 1.25	21.75 ± 2.62

**Fig. 3:**
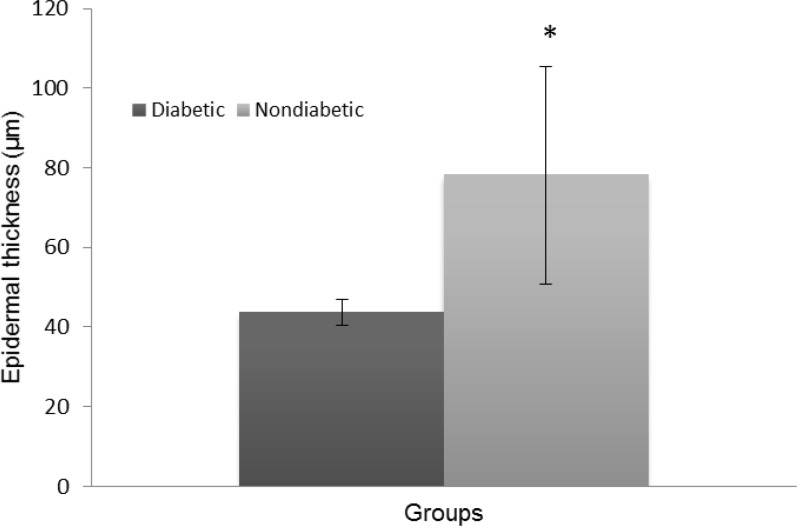
Epidermal thickness (μm) of diabetic and nondiabetic wounds (Mean ± SD). Asterisk (*) indicates significant difference (*p *< 0.05) between diabetic and nondiabetic wounds.

**Fig. 4:**
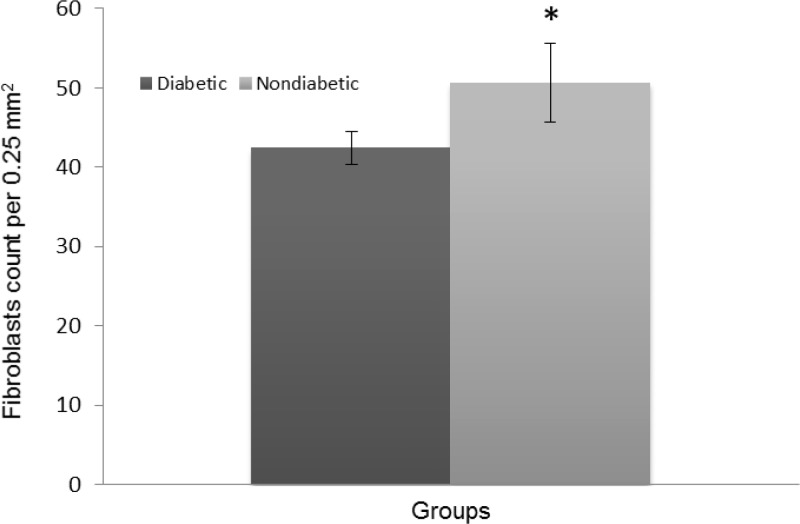
Number of fibroblasts per 0.25 mm2 (Mean ± SD). Asterisk (*) indicates significant difference (*p* < 0.05) between diabetic and nondiabetic wounds.

**Fig. 5:**
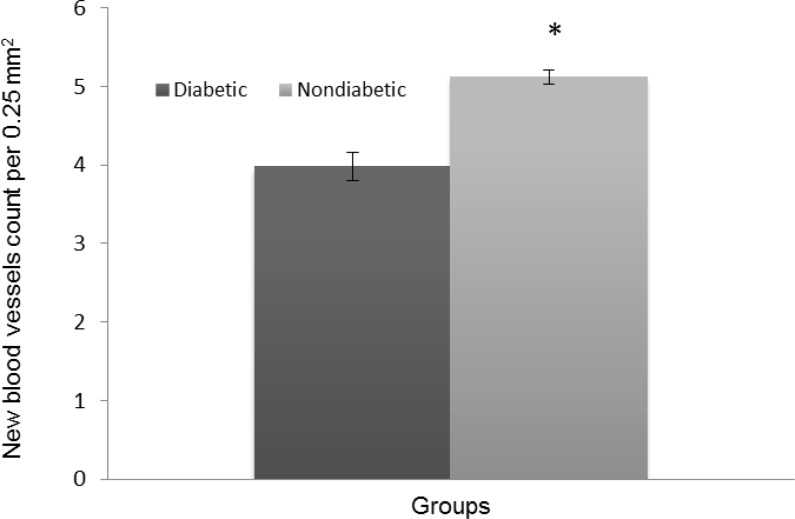
Number of new blood vessels per 0.25 mm^2^ (Mean ± SD). Asterisk (*) indicates significant difference (*p *< 0.05) between diabetic and nondiabetic wounds.

**Fig. 6:**
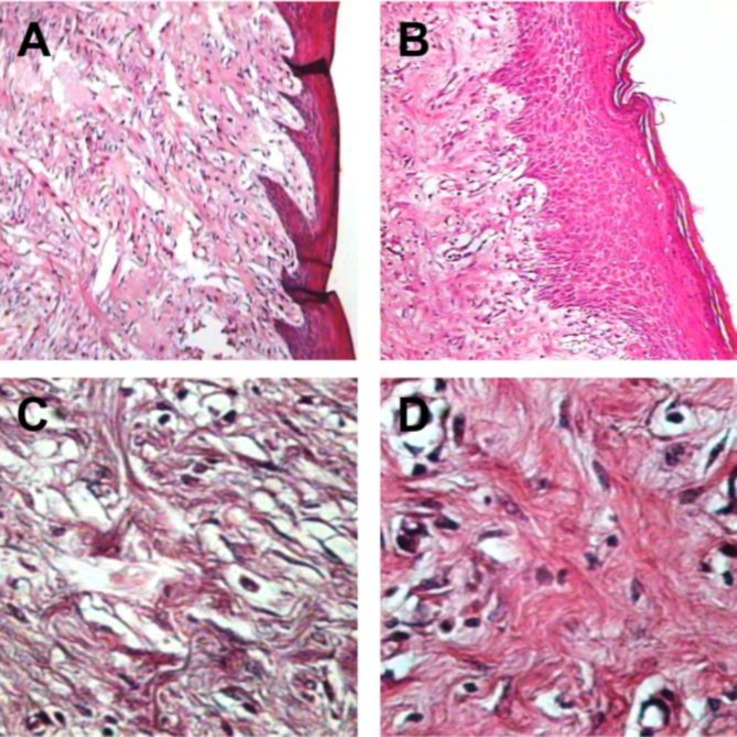
Experimental Wounds, **A)** Diabetic group. Regenerated epidermis with loose texture regenerated tissue (H&E, 100×). **B) **Nondiabetic group. Growing epidermis with the underlying granulation tissue (H&E, 100×). **C)** Diabetic group. Repaired area with fibroblasts showing loose texture (H&E, 400×). **D) **Nondiabetic group. Dense fibroblastic proliferation with blood vessels and collagen fibers (H & E, 400×).

The histopathologic parameters including epidermal thickness, fibroblast count and number of new blood vessels are expressed in figures 3, 4, and 5, respectively. All of these parameters in diabetic group were significantly lower than those of nondiabetic group (*p* < 0.05). Not only epidermis was thicker in nondiabetic group (Figs. 6A and 6B), but also fibroblast proliferation and collagen synthesis in this group were more than those of diabetic group (Figs. 6C and 6D).

## Discussion

We studied wound healing characteristics of alloxan-induced diabetes in sheep. Although diabetes develops in sheep,^19^ to the best of our knowledge, healing characteristics of excisional diabetic wound in ovine model has not been documented. The results of this study revealed impairment of wound healing process in diabetic sheep model by planimetric and histopathologic evaluations. 

Currently, the diabetic animal models used in researches include normoglycemic, oral glucose loading, chemical induction with streptozotocin or alloxan, and surgical diabetic models. Alloxan is the second most commonly used chemical for induction of diabetes mellitus.^22^ Alloxan exerts specific, irreversible effects on β-cells of pancreas leading to complete inhibition of pro-insulin biosynthesis.^23^ With this agent, it is possible to produce different grades of severity of the disease by varying the dose of alloxan used.^22^ Reportedly, injection of alloxan to sheep at the dose of 28 mg kg^-1^ has the success rate of 45.00% and mortality rate of 15.00%.^19^ In a similar study, only one of three ewes injected with alloxan at a dose of 50 mg kg^-1^ showed diabetes.^24^ Another study showed diabetes developing in all sheep at a dose of 45 mg kg^-1^ and mortality rate of 2.50%.^25^ No specific cause for such loss in alloxan-induced diabetic model has been documented, and this resistance may be also seen in other species such as rabbits.^26^

Although diabetes can develop in sheep a day after intra-venous injection,^24^ in our experience, the dose of 45 mg kg^-1^ of alloxan was not effective on two-week follow-up. However, after injection of alloxan at a dose of 60 mg kg^-1^, all of the sheep became diabetic and none of them died during the study. Alloxan is metabolized very rapidly in the body and its half-life is less than 1 min.^27^ This fact rules out the cumulative effect of first and second alloxan doses in model animals. Insulin plays a crucial role in the control of glucose metabolism. This hormone enhances glucose consumption in muscle and adipose tissues and stimulates phosphorylation of glucose and synthesis of glycogen in the liver.^28^ Although vast range of plasma insulin varying from 10 to 50 μU mL^-1^ has been reported in sheep,^29^ and the exact amount of hypoinsulinemia indicating diabetes has not been documented in this species as well. Our data showed apparent decrease of serum insulin level a week after alloxan injection and we were convinced about diabetes development. Distinct concurrent hyperglycemia also confirmed this situation. The high concentration of glucose in the wound environment promotes glycation and abnormal binding of proteins that lead to the formation of advanced glycation end-product (AGE) and oxygen free radicals. Free radicals disrupt the redox balance, damaging the DNA, and lead to poor healing.^18^ Body weight of the diabetic group was decreased during the study, but nondiabetic group showed slightly increase in body weight. The rationale for this difference may be affection of carbohydrate, protein, and fat metabolism with insulin deficiency. Thus, muscle tissue undergoes catabolic metabolism for energy, and protein synthesis is inhibited, resulting in muscle wasting.^30^

Healing of diabetic wounds in many species are relatively delayed compared with nondiabetic wounds due to vascular, neuropathic, immune, and biochemical abnormalities and are associated with high blood glucose levels.^31^^-^^33^ In the present study, wound contraction of nondiabetic group reached to 84.25 ± 3.77%, while diabetic group showed only 77.75 ± 7.76% of contraction in 21^st^ day of wounding. Nevertheless, this difference was not significant (*p *≥ 0.05). There are many metabolic differences between ruminants and other species. For instance, volatile fatty acid (VFA), and not glucose, is major source of energy for these animals, and only small amount of glucose is absorbed from the digestive tract of ruminants. Furthermore, insulin regulation of ruminants is less dependent on glucose.^28^^,^^34^^,^^35^ Thus, sheep are less responsive to insulin than non-ruminants,^36^ and our finding may be related to these differences. Since high blood glucose level is the major factor affecting wound healing in diabetes,^37^ it is presumed that wound healing in diabetic ruminants may not greatly affect because of their lower concentration of blood glucose, even in diabetic state. However, for complete and correct understanding of exact roles of these parameters, there is a need to do more studies with further samples.

Again, our histopathologic findings showed that angiogenesis, re-epithelialization, and fibroblast population of excisional diabetic wounds are significantly lower than those of normal wounds. The fewer fibroblast population in the wound, the fewer produced collagen fibers.^7^ Our findings also revealed lower density of collagen fibers along with their more irregular pattern in diabetic group than that of nondiabetic group. It means quality of healing is compromised and proliferative phase of healing is delayed in such wounds, despite nearly normal rate of wound closure. Migration of fibroblasts to the wound bed during healing process leads to granulation tissue formation and angiogenesis. Therefore, oxygen and necessary nutrients for the healing process reach readily to the wound. Some of the fibroblasts also differentiate into myofibroblasts, which will help in contraction of the wound edges.^38^ Granulation tissue formation allows migration of epithelial cells across the new tissue and the re-epithelialization takes place.^2^^,^^23^ Considering these important features of fibroblast and angiogenesis, we can presume wounds with more angiogenesis and fibroblast proliferation may heal better than others. Protease concentration is increased in diabetic wounds. These enzymes degrade tissue proteins and interrupt new extra-cellular matrix (ECM) formation. The defective scaffold with lower concentration of collagen and protein diminishes cell proliferation stimulation. On the other hand, in proliferative phase, the activity of fibroblasts is reduced and the migration of keratinocytes is impaired.^18^ In diabetic wounds, inflammatory phase of healing is pro-longed, synthesis and organization of collagen is impaired,^39^ angiogenesis is decreased,^40^ and formation of mature granulation tissue is delayed.^41^ In normal wound healing, endothelial cells of granulation tissue and epithelial cells undergo apoptosis in late phase of healing. Signals of apoptosis are also involved in the collagen degradation by inducing collagenase activity and decreasing fibroblast numbers. But in diabetic wounds apoptosis is increased throughout the healing process.^37^ This fact supports our histopathologic findings. Wound healing process can be divided into primary and secondary intentions. Healing with second intention process takes much longer time than primary intention, despite the similarity of these processes.^42^ The results of this study showed adverse effect of diabetes on second intention wound healing of sheep. Therefore, for further evaluation of diabetes effect on wound healing in ruminants, incisional wound model with primary intention healing process may be required.

In conclusion, healing of excisional diabetic wounds in sheep may be compromised, as seen in other species. However, the contraction rate of these wounds may not be delayed due to metabolic features of ruminants and these animals might go under surgeries without any serious concern. Nevertheless, more animals involvement in such studies as well as considering longer time period between diabetes induction and wounding, and using other wound models such as incisional wounds are proposed.
